# Is transcranial direct current stimulation (tDCS) effective to improve cognition and functionality after severe traumatic brain injury? A perspective article and hypothesis

**DOI:** 10.3389/fnhum.2023.1162854

**Published:** 2023-08-10

**Authors:** Bárbara Naeme de Lima Cordeiro, Elizângela Kuster, Aurore Thibaut, Lucas Rodrigues Nascimento, Jessica Vaz Gonçalves, Guilherme Peixoto Tinoco Arêas, Wellingson Silva Paiva, Fernando Zanela da Silva Arêas

**Affiliations:** ^1^Department of Physiological Sciences, Universidade Federal do Espírito Santo, Vitória, Brazil; ^2^Center of Health Sciences, Discipline of Physical Therapy, Universidade Federal do Espírito Santo, Vitória, Brazil; ^3^Coma Science Group, GIGA Consciousness, University of Liège, Liège, Belgium; ^4^Laboratory of Neurorehabilitation and Neuromodulation, Department of Physiological Sciences, Universidade Federal do Espírito Santo, Vitória, Brazil; ^5^Physiology Science Laboratory, Universidade Federal do Amazonas, Manaus, Brazil; ^6^Division of Neurosurgery, Hospital das Clinicas HCFMUSP, São Paulo, Brazil

**Keywords:** traumatic brain injury, neuromodulation, transcranial direct current stimulation, cognition, functionality, rehabilitation

## Abstract

Severe traumatic brain injury (sTBI) is an important cause of disability and mortality and affects people of all ages. Current scientific evidence indicates that motor dysfunction and cognitive impairment are the main limiting factors in patients with sTBI. Transcranial direct current stimulation (tDCS) seems to be a good therapeutic option, but when it comes to patients with sTBI, the results are inconclusive, and some protocols have not yet been tested. In addition, there is still a lack of information on tDCS-related physiological mechanisms, especially during the acute phase. In the present study, based on current evidence on tDCS mechanisms of action, we hypothesized that performing tDCS sessions in individuals with sTBI, especially in the acute and subacute phases, together with conventional therapy sessions, could improve cognition and motor function in this population. This hypothesis presents a new possibility for treating sTBI, seeking to elucidate the extent to which early tDCS may affect long-term clinical outcomes.

## 1. Introduction

Severe traumatic brain injury (sTBI) is a major cause of death and disability affecting all ages worldwide with nearly 70 million new cases each year (Maas et al., 2017). Brain injuries resulting from sTBI typically lead to cognitive, psychological, sensory, behavioral, and motor, including post-traumatic epilepsy, problems that preclude daily activities and social participation (Areas et al., 2019). TBI is largely heterogeneous and the consequences vary according to the complexity and severity of the lesions, as it may affect diverse cortical and sub-cortical brain structures (Zaninotto et al., 2019). In addition, sTBI produces high costs for the government, families, and society (Riggio, 2011).

Physical disability after sTBI (Glasgow Coma Scale 15 to 13 = mild TBI; 12 to 9 = moderate TBI; 8 45 or less = severe TBI) is considerably higher in comparison with able-bodied individuals (Lingsma et al., 2010), leading to sedentary behavior and dependence for performing activities of daily living (Williams et al., 2019). In addition, most individuals remain with cognitive deficits for example attention, memory, executive functions, language, etc. (Alderman and Wood, 2013), which is one of the main limiting factors in post-TBI patients. The diffuse axonal injury in association with secondary injuries and a cascade effect triggered by neurotoxicity explain the high incidence of cognitive impairments (Gupta et al., 2016; Stewan Feltrin et al., 2018). These chronic impairments in cognitive functions (Mcmillan and Wood, 2017; Williams et al., 2019) severely affect patients’ psychosocial recovery and may increase predisposition to neurodegenerative diseases (Alderman and Wood, 2013).

Non-invasive brain stimulation (NBIS) has been recommended for treating cognitive impairments and functional limitations in people with neurological and psychiatric disorders (Fregni and Pascual-Leone, 2007). A widely used technique is transcranial direct current stimulation (tDCS), which has the potential to modify and modulate the polarity of the neuron’s membrane potential (Nitsche et al., 2008). tDCS has been tested in several disorders (Lefaucheur et al., 2017), including TBI, (Villamar et al., 2012) based on the application of a low intensity electric current (generally 1–2 mA) for by means of two electrodes positioned on the patient’s scalp. Previous studies (Nitsche and Paulus, 2000; Boggio et al., 2008) have shown that anodal tDCS increases the excitability of the cerebral cortex and that cathodal stimulation decreases it. On a behavioral level, anodal tDCS can improve motor task performance, language, and memory. In contrast, cathodal tDCS can also increase performance by decreasing the hyperarousal in an area of maladaptive plasticity (Li et al., 2015).

Thus, depending on the size and location of the electrodes applied, tDCS may focally suppress or to modulate neuronal firing after sTBI, which may offer a promising method to minimize damage and promote functional recovery. In addition, cathodal and anodal tDCS can be used to suppress and increase, respectively, the excitability of neurotransmitters such as glutamate and GABA (gamma-aminobutyric acid) that are part of the cascade of neurochemical responses that occurs after brain trauma. Cathodal tDCS can be used to suppress acute glutamatergic hyperexcitability after sTBI. In the subacute phase, high levels of GABA can cause excessive inhibition, making recovery difficult. Therefore, modulation of GABAergic inhibition may be beneficial to minimize the functional impact at this stage. Finally, in the chronic phase of TBI, brain stimulation combined with rehabilitation can improve behavioral recovery, learning of new skills, and cortical plasticity (Demirtas-Tatlidede et al., 2012).

Previous research have demonstrated positive results after stroke (Lefaucheur et al., 2017), such as improved motor and cognitive function, when tDCS was combined with other therapies (Zaninotto et al., 2019). Besides revious studies showed evidence level B to either support the use of tDCS after acute or chronic Stroke (Elsner et al., 2017; Fregni et al., 2021). Factors related to the biological systems and individual variability are the major reasons that underlie some of these inconsistencies (Pruski and Cantarero, 2020). In addition, methodological issues related to the administration of tDCS, such as electrode montage and type, dosage, timing of application, and endpoint measures, prevent conclusions regarding its efficacy after sTBI (Arêas et al., 2022).

Studies showing positive results of tDCS after sTBI were performed in individuals in the chronic phases of trauma (Kang et al., 2012; Ulam et al., 2015; Zaninotto et al., 2019), revealing a lack of studies examining tDCS acutely after sTBI, which would be clinically relevant as studies suggest that early interventions are critical for optimal recovery (Villamar et al., 2012). According to Zaninotto et al. (2019) the combination of tDCS with cognitive and/or physical training may enhance long-term potentiation (LTP), which makes this approach very attractive, especially in acute and subacute rehabilitation settings. Therefore, this study aims to elucidate how tDCS may help improving cognition and motor function in individuals with sTBI in the acute and subacute phases, by presenting a hypothesis model.

## 2. Methods

### 2.1. Identification and selection of studies

The searches were performed in the Medline database and the reference lists of included studies were screened to identify further relevant studies. Seven randomized controlled trials that analyzed the potential effects of tDCS on brain activity in individuals with a disorder of consciousness after sTBI were included. Detailed information was provided in Table 1.

**TABLE 1:** Characteristics of included studies.

References	Study design	*N*	Age (years), mean (SD)	Time since injury	Sex (ratio F:M)	Design	Blinding	Stimulation site	Sham location	Parameters	Results
Estraneo et al., 2017	RCT	23		≥3 months after brain injury	06:07:00	Crossover	Double-blind	Anode in L-DLPFC. Cathode in the superior margin of the right orbit.	Anode in L-DLPFC. Cathode in the superior margin of the right orbit. Electrodes: 35-cm2 (7 × 5 cm)	10 sessions (5 active and 5 sham); 20 min of anodal tDCS, 2 mA; or sham tDCS. One-week washout	Negative. Substantial clinical and EEG changes were observed in 5/13 patients (3 in MCS and 2 in VS). No baseline features distinguished patients who improved from patients who did not improve.
Martens et al., 2018	RCT	22	41.86	Chronic MCS	06:16:00	Crossover	Double-blind	Anode in L-DLPFC. Cathode in the superior margin of the right orbit.	Anode in L-DLPFC. Cathode in the superior margin of the right orbit. Electrodes: 35-cm2 (7 × 5 cm)	20 sessions in two periods with an interval of 8 weeks; 20 min of anodal tDCS, 2 mA or tDCS sham.	Positive. A moderate effect size (0.47 and 0.53, for modified intention to treat and per protocol analysis, respectively) was observed at the end of the 4 weeks of tDCS in favor of the active treatment. Electrodes:
Rushby et al., 2020	RCT	30	50 (15.09)	13.9 ± 12.12 years since	09:21:00	Crossover	Single-blind	Anode in left parietal cortex.	Anode in left parietal cortex.	One session; 2 mA for 20 min.	Negative. tDCS led to no improvements in accuracy on the working memory tasks. A slight increase in variability and reaction time with tDCS was related to decreased task activated arousal. electrodes
Sacco et al., 2016	RCT	32	Experimental group: 37.7 (10.4) control group: 35.2 (12.9)	8.73 ± 4.45 years	06:26:00	Parallel groups	Double-blind	Bi-montage: anode in DLPFC right or left (the lesioned hemisphere. Cathode on the other hemisphere.	Bi-montage. (7 × 5 cm, 35 cm2)	10 sessions (twice a day); 20 min; 2 mA.	Positive. The results showed that the experimental group significantly improved in DA performance between pre- and post-treatment, showing faster reaction times (RTs), and fewer omissions.
Thibaut et al., 2014	RCT	55		1 week after acute traumatic or non-traumatic insult		Crossover	Double-blind	Anode in L-DLPFC. Cathode in the superior margin of the right orbit. the superior margin of the right orbit.	Anode in L-DLPFC. Cathode in the superior margin of the right orbit. Electrodes: 35-cm2 (7 × 5 cm)	2 sessions; 20 min of anodal tDCS, 2 mA; or sham tDCS; 48-h washout.	Negative. Patients in MCS (*n* = 30; interval 43 ± 63 months; 19 traumatic, 11 non-traumatic) showed a significant treatment effect (*p* = 0.003) as measured by CRS-R total scores. In patients with VS/UWS (*n* = 25; interval 24 ± 48 mo; 6 traumatic, 19 non-traumatic), no treatment effect was observed (*p* = 0.952). Thirteen (43%) patients in MCS and 2 (8%) patients in VS/UWS further showed post-anodal tDCS-related signs of consciousness, which were observed neither during the pre-tDCS evaluation nor during the pre- or post-sham evaluation.
Thibaut et al., 2014	RCT	21	47 (17–74)	Chronic MCS	05:09:00	Crossover	Double-blind	Anode in L-DLPFC. Cathode in the superior margin of the right orbit.	Anode in L-DLPFC. Cathode in the superior margin of the right orbit. 35-cm2 (7 × 5 cm)	10 sessions (5 active and 5 sham); 20 min of anodal tDCS, 2 mA; or sham tDCS. One-week washout sham); 20 min of anodal tDCS, 2 mA; or sham tDCS. One-week washout	Positive. A treatment effect (*p* = 0.013; effect size = 0.43) was observed at the end of the active tDCS session (day 5) as well as 1 week after the end of the active tDCS session (day 12; *p* = 0.002; effect size = 0.57).
Ulam et al., 2015	RCT	26	31.4 (35.70)	Hospitalized in the acute and subacute phase of brain injury.	04:22:00	Parallel group	Double-blind	Anode in L-DLPFC. Cathode in the superior margin of the right orbit.	Anode in L-DLPFC. Cathode in the superior margin of the right orbit. Electrodes were 3.8 cm × 4.4 cm	10 consecutive sessions; 1 mA; 20 min.	Positive. Theta was significantly reduced for active tDCS patients following the first tDCS session. Delta decreased and alpha increased, both significantly, for the active tDCS group after 10 consecutive tDCS sessions. Decreases in delta were significantly correlated with improved performance on neuropsychological tests for the active tDCS group to far greater degree than for the sham group.

L-DLPFC, left dorsolateral prefrontal cortex; L-TC, left temporal cortex; DOC, disorder of consciousness; MCS, minimally conscious state; mTBI, mild traumatic brain injury.

### 2.2. Results of the studies

The first study examined the effects of a single session of tDCS over left DLPFC cortex in 55 participants. The experimental group received 20 min of stimulation at 2 mA. The control group received a sham-stimulation. There was a significant difference in favor of the experimental group on consciousness, in people with severe brain damage (Thibaut et al., 2014).

The second study examined the effects of 10 consecutive sessions of tDCS over left DLPFC cortex in 36 participants. The experimental group received 20 min of continuous direct current stimulation at 1 mA. The control group received a sham-stimulation. There was a significant difference in favor of the experimental group in the regulation of cortical excitability (Ulam et al., 2015).

The third study examined the effects of 10 sessions of tDCS over the left DLPFC cortex (their specific location depended on participants’ damaged area) combined with computer-assisted training, 2 times per day, in 22 participants. The experimental group received 20 min of tDCS stimulation followed by 30 min of attention training. The control group received a sham-stimulation followed by 30 min of attention training. There was a significant difference in favor of the experimental group on neural reorganization and cognitive effort (Sacco et al., 2016).

The fourth study examined the effects of 5 consecutive sessions of tDCS over left DLPFC cortex in 16 participants. The experimental group received 20 min of stimulation at 2 mA. The control group received a sham-stimulation. There was a significant improvement on the recovery of consciousness in some chronic patients of the experimental group (Thibaut et al., 2017).

The fifth study examined the effects of 5 consecutive sessions of tDCS over left DLPFC cortex in 13 participants. The experimental group received 20 min of stimulation at 2 mA. The control group received a sham-stimulation. There was no difference between-groups on short-term clinical and EEG in patients with prolonged disorders of consciousness (Estraneo et al., 2017).

The sixth study examined the effects of 20 sessions of tDCS over left DLPFC cortex, 5 times per/week, during 4 weeks in 27 participants. The experimental group received 20 min of stimulation at 2 mA. The control group received a sham-stimulation. There was a significant difference in favor of the experimental group on signs of consciousness in chronic minimally conscious state patients (Martens et al., 2018).

The last study examined the effects of a single session of tDCS over left parietal cortex in 30 participants. The experimental group received 20 min of stimulation at 2 mA. The control group received a sham-stimulation. There was no difference between-groups on working memory (Rushby et al., 2020).

Overall, the experimental groups always received stimulation during 20 min at 2 mA; however, the session frequency and program duration varied across trials. The control group always received a sham-stimulation. Most trials reported positive results in favor of the experimental group, which suggests that brain stimulation has a significant effect in outcomes in individuals with disorder of consciousness after TBI. On the other hand, most studies similar methodological limitations: small sample size, heterogeneity regarding participants’ baseline conditions (i.e., diagnosis of consciousness disorders varied from months to years and varied initial level of consciousness) and medical support, lack of follow-up measurements.

## 3. Hypothesis

Studies have shown that tDCS has the potential to regulate brain function by activating the cerebral cortex using electrical current and may, therefore, induce an increase or decrease in motor cortical excitability, depending upon the polarity of tDCS (Groppa et al., 2010). In addition, tDCS increases cerebral blood volume and cerebral blood flow, and decreases the mean transit time, which suggests better oxygen delivery to cerebral tissues (Rango et al., 2008; Trofimov et al., 2018). To support this finding, imaging exams revealed areas of hyperperfusion in the basal nuclei after tDCS application, suggesting a modulatory effect on deep brain areas following tDCS application (Junior et al., 2015). Taken together, these evidences suggest that tDCS may provide benefits related to blood flow, which could help individuals with sTBI by improving hypometabolic areas and regulating those with hyperperfusion.

Our hypothesis is that tDCS applied during the acute phase in individuals with sTBI may facilitate brain plasticity. The anode may promote depolarization of the neuronal membrane and long-term potentiation, which may stimulate hypoactive brain areas. Otherwise, the cathode, which has an inhibitory function, would hyperpolarize the neuronal membrane and promote long-term depression, which may inhibit overactive brain areas (Williams et al., 2009). Because decreased cognition and motor function are the most significant impairments after sTBI, there are plausible reasons for examining the benefits of tDCS in severely disabled individuals with sTBI in the acute phase. In addition, applications focused on the left DLPF cortex in individuals with chronic TBI have already shown improvements in cognition and motor function (Li et al., 2015; O’Neil-Pirozzi et al., 2017; Li L. M. et al., 2019; Li S. et al., 2019; Quinn et al., 2020).

The integrity of white matter–mainly of the stimulated network–in TBI patients may be a factor that positively influences tDCS efficacy in such patients. A study performed by Li and colleagues that anodal stimulation improved response inhibition in control participants, an effect that was not observed in the patient group. The extent of traumatic axonal injury within the salience network strongly influenced the behavioral response to stimulation. Increasing damage to the tract connecting the stimulated right inferior frontal gyrus/anterior insula to the rest of the salience network was associated with reduced beneficial effects of stimulation. In addition, anodal stimulation normalized default mode network activation in patients with poor response inhibition, suggesting that stimulation modulates communication between the networks involved in supporting cognitive control. This is important because can show how patients with TBI can to have positive response to the tDCS approach.

One recommendation is that the severity of encephalopathy following sTBI can be objectified through neurophysiological examinations, such as electroencephalogram. To better characterize the effect of tDCS, a combination of clinical evaluations with electroencephalogram, transcranial magnetic stimulation, and/or other neurophysiological assessments may help in the development of higher quality tDCS studies in sTBI. TMS may be particularly helpful for monitoring motor responses (Zaninotto et al., 2019). In addition, these tools and high-quality imaging examinations can be useful to guide the most appropriate location for placing the electrodes.

Unfortunately, there is a lack of evidence to support or refute the hypothesis of the beneficial effects of tDCS on cognition and motor function in patients with sTBI in the acute/subacute phases (Zaninotto et al., 2019), Although some studies are in progress and show initials results (Leśniak et al., 2014; Boissonnault et al., 2021; De Freitas et al., 2021).

Besides, previous studies have suggested benefits of TMS at chronic stages after TBI (Straudi et al., 2019; Motes et al., 2020; Rushby et al., 2020), the effects of tDCS remain unclear. That is, clinical practice currently follows the do-it-yourself model, based on limited evidence (Lee and Kim, 2018; Pink et al., 2019; Nardone et al., 2020), Estimations emerge from few studies that have showed effective results on motor and cognitive impairments after stroke. We hypothesize that the addition of tDCS to motor, cognitive, or speech therapy early after sTBI would enhance potential benefits on cognition and motor function.

## 4. Discussion

Rehabilitation of individuals after sTBI has major therapeutic challenges influenced by lesion severities, phases, or symptoms. The tDCS applied to individuals with sTBI in the acute phase of the injury may have a facilitating effect on the mechanism of brain plasticity, since the anode electrode promotes depolarization of the neuronal membrane and LTP, and the cathode electrode hyperpolarizes the neuronal membrane and promotes LDP (Williams et al., 2009). In addition, early intervention may help organize the brain recovery and enhance plasticity. Furthermore, the combination of tDCS with cognitive and/or physical training may increase LTP plasticity (Rogers, 2016).

A previous systematic review revealed that new studies are needed to establish tDCS parameters such as electrode positioning, current density, duration, and stimulation intervals, as well as their effects in combination with concomitant therapies. Based on the risks of polypharmacy after sTBI, tDCS may be useful for reducing the need for drugs, or at least counteracting their cognitive side effects. In addition, improved biomarkers of neural damage due to sTBI can help understanding the mechanisms underlying the neurophysiological effects of tDCS and estimating its clinical effects and monitoring therapy. sTBI is a heterogeneous disorder and aids therapists in adapting therapy or identifying those who will most benefit. This becomes particularly challenging as sTBI is typically associated with multiple comorbidities and interventions (Zaninotto et al., 2019).

Until now, most studies after sTBI were carried out during the chronic phase (Villamar et al., 2012). There are no high-quality studies that explore the applicability of tDCS in the acute/subacute phases of sTBI aiming at improving cognitive and motor outcomes. The results of a Systematic review by Hara et al. (2021) point out that in post-stroke patients with deficits in cognitive function, including attention and memory, NIBS shows promising positive effects but this effect is limited, suggesting that further studies are needed with more precision in stimulation sites and stimulation parameters. However, results of the Systematic Review and Meta-Analysis by Ahorsu et al. (2021) showed that the overall effect of NIBS on cognition in people with TBI was moderately significant, Moreover, predictors of clinical and functional recovery after sTBI are still uncertain. These results would help elucidate the aspects linked to recovery and the use of neuromodulation within this context.

On the other hand, despite the growing interest in developing effective and innovative treatment approaches to improve outcomes after sTBI, the application of tDCS in the acute and subacute phases is challenging due to the high probability of epilepsy, unstable clinical conditions and other procedures such as craniectomy decompression and craniotomy that may limit the application of this technique (Ulam et al., 2015; Neville et al., 2019). It is worth mentioning that when it comes to acute stages of sTBI, patient interventions are very limited in clinical trials, therefore, pre-clinical studies in an animal model of acute-phase TBI as of Yoon et al. (2016) can to point stimulation currents, duration of stimulation location of electrodes and safety, to facilitate decision making from clinical trials to acute stage clinical trials in humans with severe brain injury.

## 5. Conclusion

The effects of tDCS isolated or in combination with other therapies, as well as the most appropriate doses and intensities for improving motor and cognitive outcomes early after sTBI need to be elucidated. Although preliminary studies have suggested potential effects of tDCS, the current evidence is insufficient to precisely estimate the size effects of applying tDCS in individuals with sTBI. It would be interesting to show the effects of tDCS or an estimate of its effect sizes only in the treatment of patients with TBI by performing a systematic review with meta-analysis in which a qualitative and quantitative analysis of the RCTS data is performed, we have this gap in the literature. The hypothesis raised in this manuscript by the authors and its applicability in the next studies should be carried out to investigate the effects of tDCS in the rehabilitation of TBI patients in the acute/subacute phase.

## Data availability statement

The original contributions presented in this study are included in the article/supplementary material, further inquiries can be directed to the corresponding author.

## Author contributions

All authors contributed to the development of the manuscript and read and approved the final version of the manuscript.
